# Comparison of the effects of albumin and crystalloid on mortality among patients with septic shock: systematic review with meta-analysis and trial sequential analysis

**DOI:** 10.1590/1516-3180.2017.0285281017

**Published:** 2018-10-22

**Authors:** Yan Zou, Ke Ma, Ji-Bin Xiong, Cai-Hua Xi, Xiao-Jun Deng

**Affiliations:** I MD. Associate Professor, Department of Intensive Care Medicine, Shanghai Jiao Tong University Affiliated Sixth People’s Hospital, Shanghai, China.; II MD. Associate Professor, Department of Emergency Medicine, Shanghai Jiao Tong University Affiliated Sixth People’s Hospital, Shanghai, China.; III MD. Assistant Professor, Department of Hyperbaric Oxygen Therapy, Shanghai Jiao Tong University Affiliated Sixth People’s Hospital, Shanghai, China.

**Keywords:** Albumins, Crystalloid solutions, Mortality, Shock, septic

## Abstract

**BACKGROUND:**

This study aimed to compare the effects on mortality of albumin and crystalloid, used for fluid resuscitation among adult patients with septic shock, through conducting a meta-analysis and trial sequential analysis (TSA).

**DESIGN AND SETTING:**

Meta-analysis and TSA conducted at Shanghai Jiao Tong University Affiliated Sixth People’s Hospital, Shanghai, China.

**METHODS:**

Data were collected from several major databases including MEDLINE, EMBASE, Clinical Trials.gov and Cochrane Central Register of Controlled Trials. Studies that compared the effects of albumin therapy versus crystalloid therapy on mortality among adult septic shock patients were eligible for inclusion in the analyses. The study name, year of publication, country of the trial, albumin concentration, type of crystalloid and all reported mortalities at different follow-up endpoints were extracted.

**RESULTS:**

Compared with crystalloid, albumin did not decrease all-cause mortality at the final follow-up. However, in TSA, the required information size was not achieved in all groups, which means that the effect size was not definitive and further RCTs are needed to confirm or deny these findings

**CONCLUSIONS:**

Compared with crystalloid solutions, albumin was unable to decrease all-cause mortality. However, TSA indicated that these results could be false-negative. Additional randomized controlled trials are needed to clarify this discrepancy.

## INTRODUCTION

Sepsis is a life-threatening organ dysfunction caused by a disordered response of the body to infection.^1^ Septic shock is a phenomenon relating to sepsis and is a serious disorder involving both the circulatory system and cell metabolism. During septic shock, extremely low blood pressure is observed, and this requires use of a vasoactive drug after adequate volumetric resuscitation has been applied, in order to maintain average blood pressure ≥ 65 mmHg and lactate concentration ≥ 2 mmol/l. Septic shock is the most life-threatening subtype of sepsis, with a mortality rate of 20% to 45%.^2^ Fluid resuscitation is a key component of treatments for sepsis and septic shock.

Over the past 30 years, many randomized clinical trials (RCTs) and systematic reviews^3^^,^^4^^,^^5^^,^^6^^,^^7^^,^^8^ that evaluated the therapeutic effects of various fluid resuscitation therapies on sepsis concluded that crystalloid and albumin were the most beneficial therapeutic agents, while use of artificial colloid was associated with a higher death rate and with adverse events. However, few RCTs and systematic reviews have compared the therapeutic effects of crystalloid and albumin regarding septic shock.^9^^,^^10^^,^^11^^,^^12^^,^^13^ Moreover, the researchers involved in the studies available differed in their conclusions.^14^^,^^15^^,^^16^^,^^17^^,^^18^^,^^19^^,^^20^^,^^21^

According to the findings from the Enhanced Recovery after Surgery (ERAS) study,^14^ albumin does not reduce the mortality rate due to septic shock, whereas the findings from another large RCT called ALBIOS (NCT00707122)^15^ concluded that fluid resuscitation using albumin could reduce the mortality rate from septic shock. In 2014, contrary results were reported in a meta-analysis by Patel et al.,^16^ which found that there was no difference between the effects from albumin and crystalloid treatment, while another meta-analysis by Xu et al.^17^ reported that albumin treatment had positive results with regard to reducing the mortality rate among adult patients with septic shock.

These studies have shown that it is not yet a foregone conclusion that albumin is superior to crystalloid for reducing the mortality rate in septic shock cases. In 2015, the Lactated Ringer Versus Albumin in Early Sepsis Therapy (RASP) RCT (NCT01337934)^18^ specifically compared 4% albumin and lactated Ringer’s solution with crystalloid, regarding the mortality rate among patients with septic shock. They found that resuscitation with 4% albumin, as compared with lactated Ringer, did not improve the survival rate among patients with septic shock at 30 days.

The previous descriptions show that, to date, no research findings regarding the preferred method for fluid resuscitation in septic shock cases have yet been conclusive. Our team proposed to conduct a meta-analysis focusing on the differences in the effects of albumin and crystalloid on the mortality rate due to septic shock. Moreover, we used the trial sequential analysis (TSA, available at www.ctu.tsa) method to further analyze the results from the meta-analysis. TSA is a newly proposed statistical analysis method that can improve the strength and accuracy of meta-analyses through applying an overall quantity analysis to it.

## METHODS

### Search strategy

This study was not registered. It was conducted in accordance with the guidance from the Cochrane Collaboration. The study findings were reported in accordance with the Preferred Reporting Items for Systematic Reviews and Meta-Analyses (PRISMA).^22^^,^^23^

Data were collected from the following databases: MEDLINE, EMBASE and the Cochrane Central Register of Controlled Trials (CENTRAL). The following keywords were used as searching terms: albumin, crystalloid, sepsis, pyemia*, pyohemia*, blood poisoning, mortality and survival, or prognos* and predict*. No language restrictions were placed on the search results. An additional search was carried out in Clinical Trials.gov. The date range of our search was defined as until February 27, 2017 (Table 1).

**Table 1.** Search strategies used in MEDLINE, Embase and Cochrane databases

Databases	Search strategy
MEDLINE(via PubMed)	((((“Sepsis”[Mesh]) OR (((((((sepsis[Title/Abstract]) OR septic [Title/Abstract]) OR Pyemia*[Title/Abstract]) OR Pyohemia* [Title/Abstract]) OR Pyaemia*[Title/Abstract]) OR Septicemia* [Title/Abstract]) OR Blood Poisoning[Title/Abstract]))) AND ((“Albumins”[Mesh]) OR albumin*[Title/Abstract])) AND (((“Mortality”[Mesh]) OR “Survival”[Mesh]) OR ((((((mortality [Title/Abstract]) OR fatality[Title/Abstract]) OR survival[Title/Abstract]) OR death[Title/Abstract]) OR prognos*[Title/Abstract]) OR predict*[Title/Abstract]))
EMBASE	(‘sepsis’/exp OR ( (Sepsis OR septic OR Pyemia* OR Pyohemia* OR Pyaemia* OR Septicemia*OR ‘Blood Poisoning’ ):ab,ti ) ) AND (‘albumin’/exp OR albumin*:ab,ti) AND (((mortality OR fatality OR death OR prognos* OR predict* OR survival) :ab,ti) or ‘mortality’/exp)
Cochrane Central Register of Controlled Trials	([Sepsis] or (sepsis:ti,ab,kw or sepic:ti,ab,kw or Pyemia*:ti,ab,kw or Pyohemia*:ti,ab,kw or Pyaemia*:ti,ab,kw) or (Septicemia*:ti,ab,kw or Blood Poisoning:ti,ab,kw)) and ([Albumins] or albumin*: ti,ab, kw ) and ([Mortality] or [Survival] or mortality:ti,ab,kw or survival:ti,ab,kw or fatality:ti,ab,kw or death:ti,ab,kw or prognos* :ti,ab,kw or predict*:ti,ab,kw)

### Eligibility criteria

The following types of studies were eligible for inclusion:

1. RCTs in which fluid resuscitation comparing albumin and crystalloid solution in adult patients with septic shock was studied; and
2. studies in which the mortality rate among patients with septic shock was assessed.

### Data extraction

Two reviewers (YZ and JBX) independently reviewed full-text manuscripts from the trials thus included. Any disagreement between the two reviewers was resolved through discussion or through consultation with a third reviewer (KM). Data extraction included the following: study name, year of publication, country in which the trial was conducted, trial centers, albumin concentration in the trial, type of crystalloid and all-cause mortality reported at different follow-up endpoints (CHX and XJD). The investigators also tried to contact the authors of the studies included to consult with them and clarify their data and concrete methods, when necessary (done by XJD).

### Risk of bias of studies included

The risk of bias of each study was independently assessed through using the Cochrane Risk of Bias (RoB) table,^23^ in the Review Manager (RevMan) software (version 5.3.3; Nordic Cochrane Centre, Cochrane Collaboration, Copenhagen) The RoB table considers six domains:

1. selection bias (relating to sequence generation and allocation concealment);
2. performance bias (blinding of participants and personnel);
3. measurement bias (blinding of outcome assessment);
4. loss-to-follow-up bias (any incompleteness of outcome data);
5. publication bias (freedom from selective reporting); and
6. other bias.

The overall risk of bias of individual studies was classified into the following categories: low risk of bias, unclear risk of bias and high risk of bias. Low risk was defined as a low risk of bias in all domains; an unclear risk was defined as an unclear risk of bias in at least one domain with no domains showing a high risk of bias; and high risk was defined as a high risk of bias in one or more domains. The risk of bias of the studies included is shown in Table 2.

**Table 2.** Quality evaluation on the studies included

Study name	Sequence generation	Allocation concealment	Blinding of participants and personnel	Blinding of outcome assessment	Incomplete outcome data	Selective reporting	Other bias	Overall risk of bias
Rackow et al.^19^	unclear	unclear	high risk	low risk	low risk	low risk	unclear	high risk
SAFE study^20^	low risk	low risk	low risk	low risk	low risk	low risk	unclear	unclear
EARSS study^14^	low risk	low risk	unclear	low risk	low risk	low risk	low risk	unclear
RASP study^18^	low risk	low risk	low risk	low risk	low risk	low risk	unclear	unclear
CRYSTAL study^21^	low risk	low risk	high risk	low risk	low risk	unclear	low risk	high risk
ALBIOS study^15^	low risk	low risk	unclear	low risk	low risk	low risk	low risk	unclear

### Grading the quality of evidence

The quality of evidence was assessed by means of the Grading of Recommendations, Assessment, Development and Evaluation (GRADE) methodology.^24^ The quality of evidence was classified as high, moderate, low or very low, based on judgment of the outcome of all-cause mortality, with regard to the risk of bias, inconsistency, indirectness, imprecision and other considerations.^24^ GRADE was applied firstly to each comparison of fluid resuscitation using human albumin and secondly to each predefined risk-of-bias subgroup. The summary shown in Table 3 was constructed using GRADE pro (version 3.6).

**Table 3.** Quality-of-evidence summary table according to GRADE

Quality assessment						No of patients			Effect		Quality	Importance
No of studies	Design	Risk of bias	Inconsistency	Indirectness	Imprecision	Other considerations	All-cause mortality at final follow-up after use of albumin	Crystalloid	Relative risk (95% CI)	Absolute		
All-cause mortality at final follow-up after use of albumin, compared with crystalloids												
6	randomized trials	serious^1^	no serious inconsistency	no serious indirectness	no serious imprecision	none	466/1282 (36.3%)	705/1806 (39%)	RR 0.91 (0.83 to 1)	35 fewer per 1,000 (from 66 fewer to 0 more)	Moderate	Critical
								44.6%		40 fewer per 1,000 (from 76 fewer to 0 more)		
All-cause mortality at final follow-up after use of different albumin concentrations, compared with crystalloid - hyperoncotic (20% albumin)												
2	randomized trials	no serious risk of bias	no serious inconsistency	no serious indirectness	no serious imprecision	none	339/957 (35.4%)	384/956 (40.2%)	RR 0.88 (0.79 to 0.99)	48 fewer per 1,000 (from 4 fewer to 84 fewer)	High	Critical
								38.1%		46 fewer per 1,000 (from 4 fewer to 80 fewer)		
								35.4%		18 more per 1,000 (from 92 fewer to 177 more)		
Effect of use of albumin versus crystalloid on all-cause mortality among patients with septic shock - subgroup assessed regarding mortality after 28 days (follow-up after 28 days)				
4	randomized trials	serious^1^	no serious inconsistency	no serious indirectness	no serious imprecision	none	215/717 (30%)	381/1239 (30.8%)	RR 0.96 (0.83 to 1.11)	12 fewer per 1,000 (from 52 fewer to 34 more)	Moderate	Critical
								33.7%		13 fewer per 1,000 (from 57 fewer to 37 more)		
Effect of use of albumin versus crystalloid on all-cause mortality among patients with septic shock - mortality assessed after 90 days (follow-up after 90 days)												
2	randomized trials	serious^1^	no serious inconsistency	no serious indirectness	no serious imprecision	none	265/617 (42.9%)	478/1120 (42.7%)	RR 0.89 (0.79 to 1)	47 fewer per 1,000 (from 90 fewer to 0 more)	Moderate	Critical
								42.6%		47 fewer per 1,000 (from 89 fewer to 0 more)		
All-cause mortality at final follow-up according to different risks of bias - All-cause mortality at final follow-up in subgroup with low or unclear risk of bias												
4	randomized trials	no serious risk of bias	no serious inconsistency	no serious indirectness	no serious imprecision	none	439/1216 (36.1%)	505/1245 (40.6%)	RR 0.9 (0.82 to 0.99)	41 fewer per 1,000 (from 4 fewer to 73 fewer)	High	Critical
								44.6%		45 fewer per 1,000 (from 4 fewer to 80 fewer)		
All-cause mortality at final follow-up according to different risks of bias - All-cause mortality at final follow-up in subgroup with high risk of bias												
2	no methodology chosen					none	27/66 (40.9%)	200/561 (35.7%)	RR 1.03 (0.75 to 1.42)	11 more per 1,000 (from 89 fewer to 150 more)	High	Critical
								55.2%		17 more per 1,000 (from 138 fewer to 232 more)		

### Statistical analysis

The outcomes from the trials included were pooled in terms of either relative risk (RR [risk ratio = relative risk]) for dichotomous outcomes or mean differences for continuous outcomes with 95% confidence intervals (CIs). All statistical analyses were performed using the RevMan 5.3.3 software and the Trial Sequential Analysis software (version 0.9 beta). A random-effects model (Mantel-Haenszel method) was used in the presence of statistical heterogeneity or if the situation was judged to present potential for clinical heterogeneity.^25^

**Figure 1.**
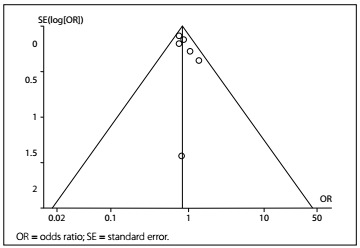
Publication bias was evaluated using a funnel plot.

Findings in which the 95% CI boundaries of TSA did not include null (< 1.00 or > 1.00) were considered statistically significant. The risk of type I error was maintained at 5% with a power of 80%. The anticipated relative risk and the event proportion in the control arm refer to the results from our meta-analysis. Publication bias was evaluated using a funnel plot (Figure 1). Rational sensitivity analysis was not conducted.

## RESULTS

### Study identification and selection

**Figure 2.**
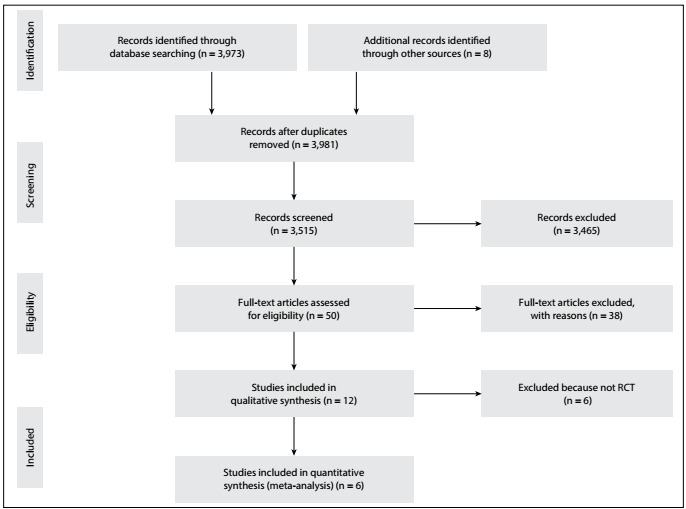
Flow chart of study selection.

A total of 3,981 records were identified in the initial search, and of these, 466 records were removed as duplicates. The remaining 3,515 records were screened. After an assessment of the titles and abstracts, 3,465 articles were excluded as not relevant to the analyses. A total of 50 studies were identified as potentially eligible for inclusion. After screening the full-text articles, 12 studies that compared albumin with crystalloid solutions among patients with septic shock were found to be eligible for inclusion. Of these, six studies did not meet the first eligibility criterion (i.e. prospective RCTs) and were excluded (Supplementary file). Thus, in the end, only six studies^14^^,^^15^^,^^18^^,^^19^^,^^20^^,^^21^ representing 3,088 patients with septic shock were included in the meta-analysis and TSA (Figure 2, flow chart). The characteristics of the studies included are listed in Table 4.

**Table 4.** Characteristics of studies included

Study name	Year	Country	Centers	Albumin concentration	Type of crystalloid	28-day mortality (albumin group)	28-day mortality (crystalloid group)	90-day mortality (albumin group)	90-day mortality (crystalloid group)	Final follow-up (albumin group)	Final follow-up (crystalloid group)
Rackow et al.^19^	1983	USA	1	5%	Normal saline	N/A*	N/A	N/A	N/A	5/7	3/4
SAFE study^20^	2004, 2011	Australia and New Zealand	16	4%	Normal saline	70/209	90/229	N/A	N/A	70/209	90/229
EARSS study^14^	2011	France	29	20%	Normal saline	96/399	103/393	N/A	N/A	96/399	103/393
CRYSTAL study^21^	2013	France, Belgium, Canada, Algeria and Tunisia	57	5% or 20%	Normal saline	19/59	157/557	22/59	197/557	22/59	197/557
ALBIOS study^15^	2014	Italy	100	20%	Crystalloid	N/A	N/A	243/558	281/563	243/558	281/563
RASP study^18^	2015	Brazil	1	4%	Lactated Ringer	30/50**	31/60**	N/A	N/A	30/50	31/60

*N/A = not applicable; **we assumed that 30 days was roughly equivalent to 28 days in the RASP study.

### Characteristics of studies included

The characteristics of the studies included are shown in Table 4. All-cause mortality at 28 days was explored in four studies.^14^^,^^18^^,^^20^^,^^21^ Ninety-day mortality rates were shown in two trials.^15^^,^^21^ Hospital discharge rates were reported in one study.^19^ Volume expansion for fluid resuscitation was done using 20% albumin in two trials;^14^^,^^15^ 4% or 5% albumin in three studies;^18^^,^^19^^,^^20^ and both concentrations in one trial.^16^ Normal saline was used as the crystalloid solution in four trials,^14^^,^^19^^,^^20^^,^^21^ and lactated Ringer’s solution was used in one trial.^18^ The remaining trial^10^ included different kinds of crystalloid products. Four trials had a low or unclear risk of bias^14^^,^^15^^,^^18^^,^^20^ and two studies had a high risk of bias.^14^^,^^16^

### All-cause mortality at different follow-ups after use of albumin, compared with crystalloid

#### Meta-analysis

**Figure 3.**
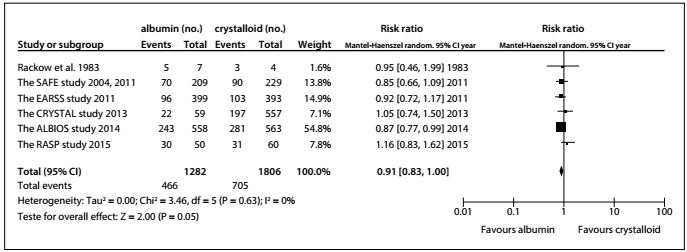
All-cause mortality ascertained at final follow-up, after use of albumin, compared with crystalloid.

**Figure 4.**
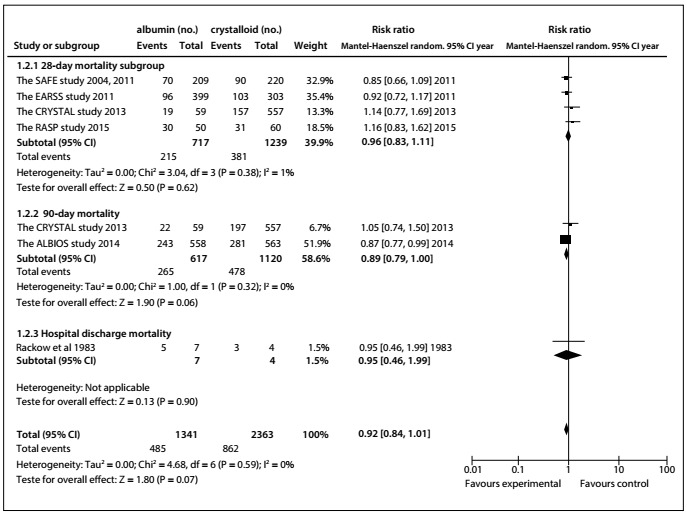
All-cause mortality ascertained at different follow-ups, after use of albumin, compared with crystalloid.

Compared with crystalloid solutions, human albumin showed no benefit regarding all-cause mortality at the final follow-up (RR: 0.91; 95% CI: 0.83-1.00; P = 0.05; I^2^ = 0%; Figure 3). Similarly, use of albumin was not found to have decreased 28-day mortality^14^^,^^18^^,^^20^^,^^21^ (RR 0.96; 95% CI: 0.83-1.11; I^2^ = 1%) or 90-day^18^^,^^21^ mortality (RR: 0.89; 95% CI: 0.79-1.00; P = 0.06; I^2^ = 0%) (Figure 4).

#### TSA

**Figure 5.**
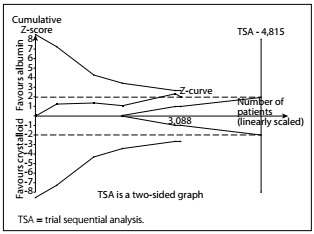
A diversity-adjusted information size of 4,815 patients was calculated using α = 0.05 (two-sided), β = 0.20 (power 80%), D2 = 0%, an anticipated relative risk of 10.0% (refer to our meta-analysis) and an event proportion of 39% in the control arm (refer to our meta-analysis). The cumulative z curve was constructed using a random-effects model. Trial sequential analysis showed that 3,088 patients out of the required information size of 4,815 patients were accrued. The cumulative z curve touched the conventional boundary for benefit but did not cross the trial sequential monitoring boundary for benefit.

A diversity-adjusted information size of 4,815 patients was calculated using α = 0.05 (two-sided), β = 0.20 (power 80%), D2 = 0%, an anticipated RR of 10.0% (Table 3) and an event proportion of 39% in the control arm (Table 3). The cumulative z curve was constructed using a random-effects model. TSA showed that, out of the required sample size of 4,815 patients, a sample size of 3,088 patients was accrued. The cumulative z curve touched the conventional boundary for benefit but did not cross the trial sequential monitoring boundary for benefit (Figure 5). This outcome indicates that the result was possibly a false negative because the required sample size was not met.

### All-cause mortality at final follow-up after use of different concentrations of albumin, compared with crystalloid

#### Meta-analysis

**Figure 6.**
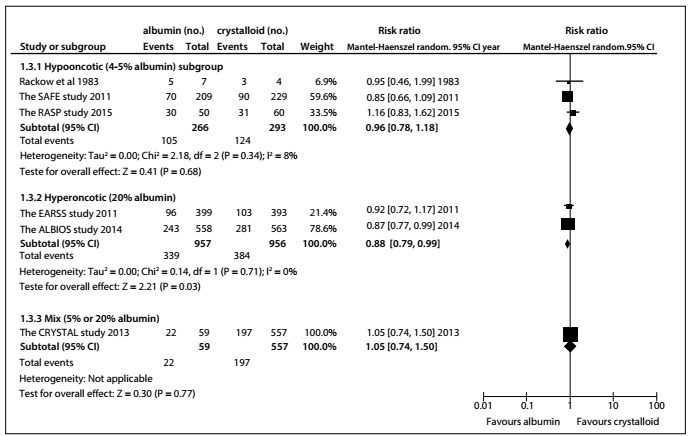
All-cause mortality ascertained at final follow-up, after use of different albumin concentrations, compared with crystalloid.

Compared with crystalloids, low concentrations of albumin (4%-5%)^18^^,^^19^^,^^20^ were not found to have reduced all-cause mortality at the final follow-up (RR: 0.96; 95% CI: 0.78-1.18; P = 0.68; I^2^ = 8%). The high concentration (20%) albumin subgroups^14^^,^^21^ were found to have slightly reduced all-cause mortality (RR: 0.88, 95% CI: 0.79-0.99, P = 0.03; I^2^ = 0%) (Figure 6).

#### TSA

**Figure 7.**
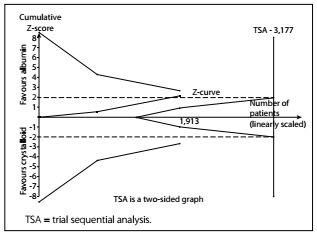
A diversity-adjusted information size of 3,177 patients was calculated using α = 0.05 (two-sided), β = 0.20 (power 80%), D2 = 0%, an anticipated relative risk of 12.0% (refer to our meta-analysis) and an event proportion of 40.2% in the control arm (refer to our meta-analysis). The cumulative z curve was constructed using a random-effects model. Trial sequential analysis showed that 1,913 patients out of the required information size of 3,177 patients were accrued. The cumulative z curve touched the conventional boundary for benefit but did not cross the trial sequential monitoring boundary for benefit.

A diversity-adjusted information size of 3,177 patients was calculated using α = 0.05 (two-sided), β = 0.20 (power 80%), D2 = 0%, an anticipated RR of 12.0% (Table 3) and an event proportion of 40.2% in the control arm (Table 3). The cumulative z curve was constructed using a random-effects model. TSA showed that, out of the required 3,177 patient sample size, only a sample size of 1,913 was accrued. The cumulative z curve touched the conventional boundary for benefit but did not cross the trial sequential monitoring boundary for benefit (Figure 7). This outcome indicates that the result was possibly a false positive because the required sample size was not met.

Funnel plots for the comparisons of human albumin with crystalloid showed that there was no publication bias. The odds ratio (OR) and its standard error (SE) are plotted in Figure 1.

## DISCUSSION

The purpose of this research was to compare the effects of albumin and crystalloid solution on the mortality rate among patients with septic shock. Meta-analysis for all-cause mortality showed that albumin offered no benefit in comparison with other interventions for septic shock patients at the final follow-up (P = 0.05). Through the subgroup analysis, significant benefits were observed for the subgroup of 20% albumin (P = 0.03). However, after conducting TSA on this meta-analysis we found that these results may not be definitive. Because the required information sizes were not reached, and because the cumulative z scores did not reach the trial sequential monitoring boundaries of benefit (Figure 5, Figure 7), these results indicated that albumin was not beneficial or harmful in these groups or subgroups. The initial meta-analysis results may have been false negative or positive outcomes. Therefore, additional high-quality RCTs are recommended in order to ensure that correct conclusions are reached. The main difference between the present analysis and previous studies is that, along with inclusion of the most recent RCTs,^14^^,^^18^ a TSA was included in the analysis to confirm the results from the initial meta-analysis.

As is well known, sample sizes need to be estimated such that clinical trials are repeatable and sufficient statistical power is ensured. In systematic reviews and meta-analyses, when the number of trials included or the total sample size is too small, the effect will be exaggerated due to random errors. TSA is a newly proposed statistical analysis method that can improve the strength and accuracy of meta-analyses through applying an overall quantity analysis to it. Duplication of a statistically significant experiment would increase the risk of type I error in the hypothesis test, which would mean creation of a false positive.^26^^,^^27^^,^^28^^,^^29^ TSA^30^ involves the principle and method of the trial sequence. Through checking the P-value and its CI, this method has the following significant advantages:

1. the conclusion can be generated earlier without increasing the type I error;
2. the sample size can be estimated; and
3. hints for further research studies are provided through numerical data and visualized sample sizes.

Research studies have shown that 25% of traditional meta-analyses produced false positive results due to small sample sizes.^31^ With the help of this advanced method, our research was more precise and novel.

From a theoretical point of view, albumin is an ideal resuscitation fluid for treating septic shock, but its use in research studies has not demonstrated it to be superior to other resuscitation fluids. The reason for this discrepancy may have been the insignificant reduction in the mortality rate when albumin was compared with crystalloids and the lack of high-quality RCTs comparing albumin and crystalloid solutions in septic shock cases. In addition, further suggestions were provided for the experimental design of further studies based on the present research. More benefits could be produced through using crystalloid solutions compared with albumin, especially at high albumin concentrations, as well as through well-designed RCTs.

There are other limitations to the present research study. Firstly, a measurable error is generated if a blinded method was not applied in the studies, and therefore it is recommended that a blinded method should be used in future research studies for better quality. Secondly, although statistical heterogeneity between studies was not found, clinical and methodological heterogeneity may have been present. Thirdly, different follow-up durations were used, and different follow-up durations would obviously have different results. Fourthly, TSA had limitations, given that it was unable to resolve the error that was generated by the quality of the initial search methodology and by the uncertainty from the result. In addition, the initial search would have affected the TSA output if its quality was low.

Although TSA showed that there was a possibility that, without inclusion of further RCTs to provide additional support, the results from the meta-analysis could have been false positives, the output from the meta-analysis was still useful as guidance for experimental designs and field applications in the future. As the results showed, there is a high possibility that a high concentration of albumin can produce a positive result regarding decreased mortality, when used for fluid resuscitation in cases of septic shock. In short, TSA should be strongly recommended and should be more used for future review studies, so that evidence of greater reliability and consistency can be obtained.

PRECISE (NCT0019416)^9^ has been completed. This is another large-scale RCT that focuses on comparing the effects of albumin and normal saline on the mortality rate due to septic shock. The conclusion of this RCT is eagerly awaited, given that the sample size of the meta-analysis will reach the required information size. Thus, it will help determine whether the reduction in mortality that is associated with use of albumin is a truly positive result or a type I error.

## CONCLUSIONS

The results from the meta-analysis showed that, in comparison with use of crystalloid, human albumin did not decrease all-cause mortality, as evaluated at the final follow-up. The trial sequential analysis results suggest that the negative results observed so far might not be definitive. Further RCTs are needed to confirm this result.

## Supplementary file.

Studies excluded and reasons for this.

**Table:**

First author	Subject	Reason for exclusion
Dahn 1979^1^	Negative inotropic effect of albumin resuscitation for shock.	Seriously injured patients were included in the study, and data on severe sepsis were inadequate.
Lucas 1979^2^	Impaired salt and water excretion after albumin resuscitation for hypovolemic shock.	Hypovolemic shock patients were included in the study, and data on severe sepsis were inadequate.
Lucas 1980^3^	Impaired pulmonary function after albumin resuscitation from shock.	Shock patients were included in the study, and data on severe sepsis were inadequate.
Brown 1988^4^	Effect of albumin supplementation during parenteral nutrition on hospital morbidity.	Patients requiring central total parenteral nutrition were included in the study, and data on severe sepsis were inadequate.
Foley 1990^5^	Albumin supplementation in critically ill patients.	Hypoalbuminemic patients were included in the study, and data on severe sepsis were inadequate.
Younes 1992^6^	Hypertonic solutions for treating hypovolemic shock: a prospective, randomized study on patients admitted to the emergency room.	Hypovolemic shock patients were included in the study, and data on severe sepsis were inadequate.
Stockwell 1992^7^	Colloid solutions in critically ill patients: a randomized comparison of albumin and polygeline. Outcome and duration of stay in the intensive care unit.	All patients were included in the study, and data on severe sepsis were inadequate.
Tuchschmidt 1992^8^	Elevation of cardiac output and oxygen delivery for improvement of outcomes in septic shock cases.	Septic shock patients were treated using an algorithm to increase confidence intervals to a different level.
Golub 1994^9^	Efficacy of albumin supplementation in surgical intensive care unit: a prospective, randomized study.	The study was conducted in the surgical intensive care unit, and data on severe sepsis were inadequate.
Steltzer 1994^10^	Hemodynamic evaluation during small-volume resuscitation among patients with acute respiratory failure.	Acute respiratory failure patients were included in the study, and data on severe sepsis were inadequate.
Boldt 1996^11^	Effects of albumin versus hydroxyethyl starch solution on cardio respiratory and circulatory variables in critically ill patients.	Comparison was between albumin and low-molecular weight hydroxyethyl starch solution, not between albumin and crystalloid.
Rock 1997^12^	Pentastarch instead of albumin as replacement fluid for therapeutic plasma exchange.	Comparison was between albumin and pentastarch, not between albumin and crystalloid.
Rubin 1997^13^	Randomized, double blind study on intravenous human albumin in hypoalbuminemic patients receiving total parenteral nutrition.	Hypoalbuminemic patients were included in the study, and data on severe sepsis were inadequate.
Ernest 1999^14^	Distribution of normal saline and 5% albumin infusions among septic patients.	Septic patients were included in the study, and data on severe sepsis were inadequate.
Wu 2001^15^	Hemodynamic response of modified fluid gelatin compared with lactated Ringer’s solution for volume expansion in emergency resuscitation of hypovolemic shock patients: preliminary report on a prospective, randomized trial.	Hypovolemic shock patients were included in the study, and data on severe sepsis were inadequate.
Oliveira 2002^16^	Acute hemodynamic effects of a hypertonic saline/dextran solution in stable patients with severe sepsis.	Comparison was between hypertonic saline/dextran solution and saline, not between albumin and crystalloid.
Quinlan 2004^17^	Albumin influenced total plasma antioxidant capacity favorably in patients with acute lung injury.	Acute lung injury patients were included in the study, and data on severe sepsis were inadequate.
Veneman 2004^18^	Human albumin and starch administration in critically ill patients: a prospective randomized clinical trial.	Severe sepsis patients were included in the study, and the mortality data on the albumin group and crystalloid group were inadequate.
Vincent 2005^19^	Albumin administration in acutely ill patients in relation to increased mortality :results from the SOAP study.	Not a randomized controlled or parallel clinical trial.
Palumbo 2006^20^	Effects of hydroxyethyl starch solution on critically ill patients.	Comparison was between albumin and hydroxyethyl starch, not between albumin and crystalloid.
Dubois 2006^12^	Albumin administration improved organ function in critically ill hypoalbuminemic patients: a prospective, randomized, controlled, pilot study.	Hypoalbuminemic patients were included in the study, and data on severe sepsis were inadequate.
Bellomo 2006^22^	Effects of saline or albumin resuscitation on acid-base status and serum electrolytes.	Not a randomized controlled or parallel clinical trial.
McIntyre 2007^23^	Resuscitating patients with early severe sepsis: a Canadian multicenter observational study.	Not a randomized controlled or parallel clinical trial.
Guidet 2007^24^	The COASST study: cost-effectiveness of albumin in cases of severe sepsis and septic shock.	Not a randomized controlled or parallel clinical trial.
McIntyre 2008^25^	Fluid resuscitation in management of early septic shock (FINESS): a randomized controlled feasibility trial.	Comparison was between pentastarch and saline, not between albumin and crystalloid.
Friedman 2008^26^	Hemodynamic effects of 6% and 10% hydroxyethyl starch solutions versus 4% albumin solution in septic patients.	Comparison was between hydroxyethyl starch and albumin, not between albumin and crystalloid.
Schortgen 2008^27^	Risk associated with hyperoncotic colloids in patients with shock.	Not a randomized controlled or parallel clinical trial.
Dolecek 2009^28^	Therapeutic influence of 20% albumin versus 6% hydroxyethyl starch on extravascular lung water in septic patients: a randomized controlled trial.	Comparison was between hydroxyethyl starch and albumin, not between albumin and crystalloid.
Bellomo 2009^29^	Effects of saline or albumin resuscitation on standard coagulation tests.	Not a randomized controlled or parallel clinical trial.
van der Heijden 2009^30^	Crystalloid or colloid fluid loading and pulmonary permeability, edema, and injury in septic and non-septic critically ill patients with hypovolemia.	Hypovolemic septic patients were included in the study, and the mortality data on the albumin group and crystalloid group were inadequate.
Trof 2010^31^	Greater cardiac response of colloid than saline fluid loading in septic and non-septic critically ill patients with clinical hypovolemia.	Critically ill septic patients were included in the study, and the mortality data on the albumin group and crystalloid group were inadequate.
Finfer 2010^32^	Resuscitation fluid use in critically ill adults: an international cross-sectional study in 391 intensive care units.	Not a randomized controlled or parallel clinical trial.
Zhu 2011^33^	Hypertonic saline and hydroxyethyl starch for treating severe sepsis.	Comparison was between hypertonic saline and hydroxyethyl starch, not between albumin and crystalloid.
Crystalloid versus Hydroxyethyl Starch Trial (CHEST) Management Committee 2011^34^	Crystalloid versus hydroxyethyl starch trial: protocol for a multicenter randomized controlled trial on fluid resuscitation with 6% hydroxyl starch (130/0.4) compared with 0.9% sodium chloride (saline) in intensive care patients, regarding mortality.	Comparison was between crystalloid and hydroxyethyl starch, not between albumin and crystalloid.
Scandinavian Critical Care Trials Group 2011^35^	Comparing the effect of hydroxyethyl starch 130/0.4 with balanced crystalloid solution on mortality and kidney failure in patients with severe sepsis (6S: Scandinavian Starch for Severe Sepsis/Septic Shock trial): study protocol, design and rationale for a double-blinded, randomized clinical trial.	Comparison was between crystalloid and hydroxyethyl starch, not between albumin and crystalloid.
McIntyre 2012^36^	Fluid resuscitation with 5% albumin versus normal saline in early septic shock: a pilot randomized, controlled trial.	Septic shock patients were included in the study, and the mortality data on the albumin group and saline group were inadequate.
van Haren 2012^37^	Hypertonic fluid administration in patients with septic shock: a prospective randomized controlled pilot study.	Comparison was between hypertonic fluid and isotonic fluid, not between albumin and crystalloid.
Myburgh 2012^38^	Hydroxyethyl starch or saline for fluid resuscitation in intensive care.	Comparison was between hydroxyethyl starch and saline, not between albumin and crystalloid.
Yunos 2012^39^	Association between a chloride-liberal vs chloride-restrictive intravenous fluid administration strategy and kidney injury in critically ill adults.	Not a randomized controlled or parallel clinical trial
McIntyre 2012^40^	The PRECISE RCT: evolution of an early septic shock fluid resuscitation trial.	Septic shock patients were included in the study, and the mortality data on the albumin group and saline group were inadequate.
Perez 2013^41^	Intravenous 0.9% sodium chloride therapy did not reduce length of stay of alcohol-intoxicated patients in the emergency department: a randomized controlled trial.	Acute alcohol intoxication patients were included in the study; the mortality data on the albumin group and saline group were inadequate; and the comparison was not between albumin and crystalloid.
Masson 2014^42^	Presepsin (soluble CD14 subtype) and procalciton in levels for mortality prediction in sepsis: data from the Albumin Italian Outcome Sepsis trial.	Comparison was not between albumin and crystalloid.
Caironi 2015^43^	Albumin in critically ill patients: the ideal colloid?	Not a controlled or parallel clinical trial.
Chang 2016^44^	Choice of fluid therapy in the initial management of sepsis, severe sepsis, and septic shock.	Choice of fluid therapy in the initial management of sepsis, severe sepsis, and septic shock.
